# Alginate-, Carboxymethyl Cellulose-, and κ-Carrageenan-Based Microparticles as Storage Vehicles for Cranberry Extract

**DOI:** 10.3390/molecules25173998

**Published:** 2020-09-02

**Authors:** Marta Tsirigotis-Maniecka

**Affiliations:** Department of Engineering and Technology of Chemical Processes, Wrocław University of Science and Technology, Wybrzeże Wyspiańskiego 29, 50-370 Wrocław, Poland; marta.tsirigotis@pwr.edu.pl; Tel.: +48-71-320-3849

**Keywords:** hydrogel, encapsulation, long-term storage, antioxidant activity, *Vaccinium macrocarpon* Aiton, fruit extract

## Abstract

This study discusses the relationship between the structural properties of the selected polysaccharides (low (ALG_LV_) and medium viscosity (ALG_MV_) sodium alginate, 90 kDa (CMC_90_) and 250 kDa (CMC_250_) carboxymethyl cellulose, and κ-carrageenan (CAR_κ_)) and their abilities to serve as protective materials of encapsulated large cranberry (*Vaccinium macrocarpon* Aiton) fruit extract (CE) from losing its health beneficial activities during long-term storage. The microparticles were characterized in terms of their encapsulation efficiency (UV-Vis and FTIR), morphology (SEM) and the physical stability in various environments (gravimetry). The microparticles’ size and encapsulation efficiency were 46–50 µm and 28–58%, respectively, and the microparticles were physically stable. CMC_90_ and ALG_MV_ most efficiently protected the plant extract from losing its biological activity after 18 months, while the plant extract stored outside the particles had lost its activity. CE was intended for oral administration, thus CE release from the microparticles was monitored in vitro under gastrointestinal conditions. In vitro gastrointestinal release studies revealed that the ALG_MV_-, CMC_90_-, and CMC_250_-based particles exhibited the desired intestinal release pattern. This result supports the suitability of sodium alginate and carboxymethyl cellulose for the safe delivery of CE to the intestines while maintaining its biological properties and improving long-term storage stability.

## 1. Introduction

Polysaccharides are the most promising materials in the biomedical field because they are biodegradable, biocompatible, and generally recognized as safe for direct human contact. Additionally, these biomaterials are derived from renewable resources and thus are easily available at a rather low cost [1]. Furthermore, the use of polysaccharides for encapsulation has been indicated to be convenient and versatile for industrial applications. Polysaccharides may be employed to fabricate effective carriers designed to deliver and release drugs in a rate-controlled and targeted manner, i.e., to enhance the bioavailability of active agents. This property is attributed to the easy functionalization of carbohydrates, and their physicochemical properties are easily tunable in terms of usage in multiple pharmaceutical dosage forms, such as drug delivery systems (DDSs), in the form of hydrogel particles for oral, intranasal, intrapulmonary, and intravenous administration [2]. Polysaccharides prevent uncontrolled mass transfer between the pharmaceutical formulation and its surroundings and form a protective barrier, which improves the stability of the pharmaceutical and extends its shelf life [3]. Among the numerous polysaccharides, alginates, carboxymethyl cellulose, and carrageenans have attracted considerable attention. Alginates (ALGs) are composed of alternating β-d-mannuronic acid (M) and α-l-guluronic acid (G) units. The performance of the selected ALGs is directly related to their molecular weight (Mw) and M/G ratio [4]. Carboxymethyl cellulose (CMC) is an ionic ether of cellulose with a carboxymethyl group substituted for hydrogen in the hydroxyl group of D-glucose units [5]. CMCs differ in terms of their degree of substitution (DS) and Mw, as reflected in their chemical compositions. Both of these properties may significantly alter the functionality of the polysaccharide. Finally, κ-carrageenan, a sulfated galactan, is composed of β-d-galactopyranose and α-d-galactopyranose units with one sulfate group per disaccharide [6]. Due to the diverse chemical compositions of these polysaccharides, the correlation between the type of polysaccharide used as the core material of a microcarriers and the functional properties of these microcarriers must be thoroughly investigated.

Because of the numerous health benefits and disease-preventing functions of phytochemicals, these molecules have attracted increasing attention from consumers. Plant preparations are advantageous, but at the same time, they are often unstable and susceptible to chemical or microbiological degradation, particularly when subjected to harsh technological processes, transportation, and storage, all of which lead to a loss of bioactivity [7]. In particular, long-term storage imposes some challenges, as phytochemicals are negatively affected by physicochemical factors (light, heat, oxygen, and humidity), resulting in changes in their chemical composition that adversely affect their biological activities [8]. One of the promising approaches to increase the stability of fragile, heat- and light-sensitive plant-derived preparations is encapsulation in a carrier. As a result of this process, polysaccharide microcarriers protect phytochemicals from a loss in biological activity, followed by targeted delivery to specific sites in the human body [9,10].

Fruits of large cranberry and their preserves (juices, infusions, teas, and pomace) are readily consumed as functional food due to their broad spectrum of properties that are beneficial for health, such as reduced risk of cancers [11] and cardiovascular diseases [12], general antimicrobial activity [13], and specific antimicrobial activity towards pathogens in the urinal tract (inhibits bacterial adhesion) [14], as well as antimutagenic, anti-inflammatory, and anti-hypercholesterolemic activities [15]. Additionally, large cranberry fruit juice has been used to treat digestive tract disorders [11]. Moreover, large cranberry fruits and fruit extracts are considered a natural source of antioxidants [16,17] that reduce cellular oxidative stress and improve resistance to H_2_O_2_-induced DNA damage [18]. Cranberry fruit extracts are abundant in organic acids (i.e., malic, citric, quinic, and benzoic acids), saccharides (i.e., glucose and fructose), and phenolic compounds, which are considered the major compounds responsible for the therapeutic properties. Phenolic compounds mainly occur in cranberry fruits in the forms of acids (i.e., hydroxycinnamic acid), flavonoids (i.e., anthocyanins, flavonols, and flavanols), and low molecular weight phenolic glycosides (i.e., cyanidin- and peonidin-3-*O*-galactoside, cyanidin- and peonidin-3-*O*-arabinosides, quercetin- and myricetin-3-*O*-galactoside, and quercetin-3-*O*-pentosides) [19]. The antioxidant mechanism of large cranberry fruit extracts has consistently been attributed to the interdependent actions of organic acids, carbohydrates, and phenolics [16,17]. Due to its multiple advantageous properties, a cranberry fruit extract was used as a model payload for polysaccharide-based microparticles developed to maintain the beneficial properties of phytopharmaceuticals during storage.

This study discusses the relationship between the structural properties of the selected polysaccharides and their abilities to serve as protective materials for the long-term storage of encapsulated, biologically active plant extracts. Five hydrophilic, gel-forming, anionic polysaccharides with various chemical compositions (low (ALG_LV_) and medium (ALG_MV_) viscosity sodium alginate, 90 kDa (CMC_90_) and 250 kDa (CMC_250_) carboxymethyl cellulose, and kappa carrageenan (CAR_κ_)) served as the core materials for the preparation of microparticles using an emulsification approach coupled with ionic gelation. The differences in the chemical structures resulted in the production of microcarriers with various features and functional properties. The effect of the polysaccharide structure on the resulting microparticles loaded with large cranberry (*Vaccinium macrocarpon* Aiton) fruit extract (CE) was comparatively investigated in terms of the physicochemical properties of the particles (size, surface microstructure, and shape), their physical stability under various conditions, and encapsulation efficiency. Changes in the antioxidant activity of the extract stored inside (CE) and outside (CES) the particles were also investigated. Finally, the abilities of the polysaccharides to control the release behavior of the payload were verified in vitro under conditions mimicking the conditions in the gastrointestinal tract. The results provide information on whether the selected polysaccharides are useful for preparing micro-delivery systems that maintain the stability of a plant extract for at least 18 months, thus extending its shelf life. Additionally, the results indicate which of the utilized polysaccharides is most compatible with CE. This study is a continuation of current research focused on developing multipurpose, biopolymeric hydrogel microcarriers designed to prevent the loss of the nutritional and functional potential of phytopharmaceuticals [20,21].

## 2. Results and Discussion

Five plant-derived carbohydrates were selected because they possess multiple functional groups that undergo ionic crosslinking and are capable of forming physically stable gels in the form of micro-sized particles, namely, 90 kDa carboxymethyl cellulose (CMC_90_) that formed SCMC, 250 kDa carboxymethyl cellulose (CMC_250_) that formed LCMC, low viscosity sodium alginate (ALG_LV_) that formed LALG, medium viscosity sodium alginate (ALG_MV_) that formed MALG, and κ-carrageenan (CAR_κ_) that formed κCAR (refer to Table 1).

The CE-loaded polysaccharide-based microparticles were fabricated using an emulsification approach coupled with an external ionotropic gelation. This technique involved the formation of a water-in-oil emulsion, where the water phase (W) was composed of CE suspended in the polysaccharide solution and the oil phase (O) was composed of liquid paraffin and an oil-soluble emulsifier. The obtained W/O emulsion containing biopolymeric droplets dispersed in paraffin oil was rapidly transferred to a large volume of agitated crosslinking medium. Following this step, the W/O emulsion was diluted with an excess of the crosslinking solution. Under the action of intense shear forces during the agitation, the droplets of polysaccharide solution passed into the aqueous solution of the crosslinker and the solidification of the microdroplets began. First, surface gelation occurred, and then crosslinking ions diffused through the volume of the microparticles towards their core [22] during the curing phase (90 min), transforming biopolymeric droplets into completely gelled microparticles in the form of a solid precipitate.

### 2.1. Characteristics of the Microparticles Depending on the Core Polysaccharide

**Figure 1:**
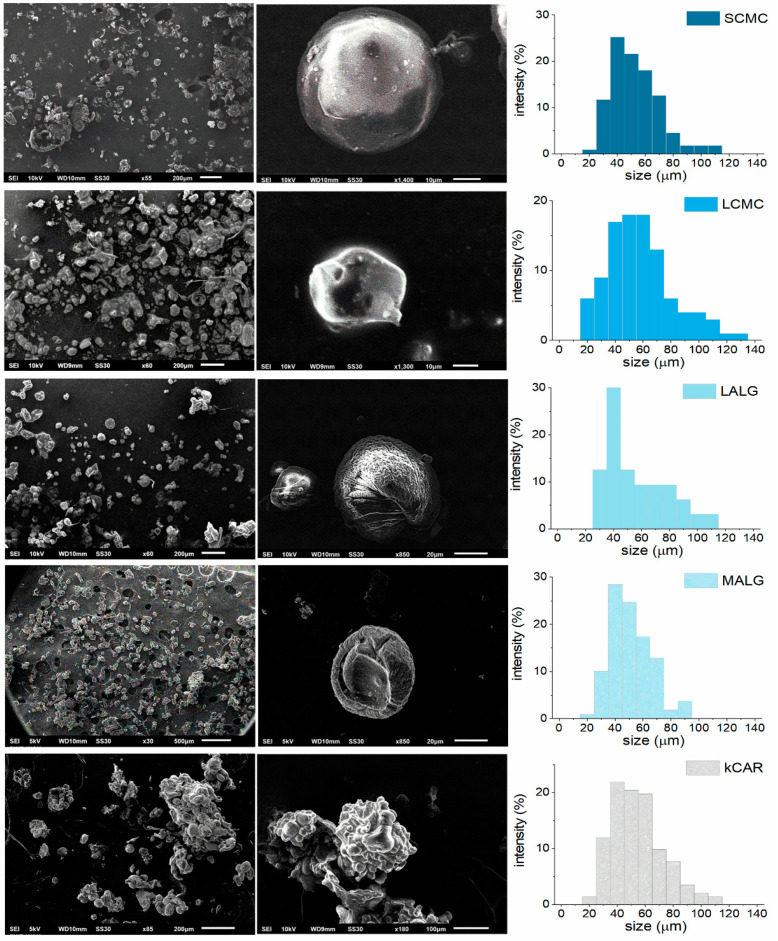
Scanning electron microscopy images and size distribution of the cranberry extract (CE)-loaded polysaccharide microparticles.

The diverse molecular weights and densities of the functional groups of the selected polysaccharides resulted in the production of hydrogels with different structures due to their various water absorption abilities, which clearly altered the consistency, morphology, and encapsulation ability of the CE-loaded microparticles. CE was used as a model payload with known biological properties. The morphology of the polysaccharide microparticles was studied using scanning electron microscopy (SEM), and the SEM micrographs are presented in Figure 1.

The microparticle fabrication procedure resulted in products from a spherical-like to shapeless morphology. Due to the mechanism of microparticle formation, only a part of the particles retained their original shape resembling spheres. The SEM methodology required microparticles to be dried to remove the water. Unfortunately, drying caused shrinking and a slight change in the shape of the fabricated ALG- and CMC-based microparticles. Meanwhile, particles composed of CARκ were not only shapeless, but also aggregated, probably due to the coalescence and aggregation events occurring during the solidification step. The surfaces of the microparticles differed considerably, but no cracks or visible pores were observed. The CMC-based microparticles exhibited smooth surfaces, the ALG-based microparticles were rugged and wrinkled, whereas the surfaces of the CAR_κ_-based microparticles appeared clumpy and flaky. These differences might be attributed to the effect of the partial solidification of the κCAR particles during the formation process. The rheological behavior of the oil phase (high viscosity) during the emulsification process prevented the uniform homogenization of the CE-loaded polymer solution, leading to a reduction in the local temperature and uncontrolled CARκ solidification. Additionally, the aggregation of κCAR may be attributed to a destabilization effect caused by the incorporation of CE. The studied polysaccharide microparticles had similar sizes (46–56 µm), but the standard deviation (S.D.) values displayed a large size distribution, regardless of the type of polysaccharide matrix (Table 1).

The large size distribution is probably due to the high viscosity of the environment present during the formation of the microparticles. The homogeneous distribution of the CE-loaded polysaccharide solution was prevented in the oil phase; therefore, the size of the formed particles differed. Successful incorporation of CE in SCMC, LCMC, LALG, MALG, and κCAR was confirmed by the results of the Fourier transform infrared (FTIR) spectroscopy analyses (Figure S2, Supplementary Materials) (the explanation is included in the Supplementary Materials) [23]. The CE encapsulation efficiency (EE) of the ALG- and CMC-based microparticles was within the acceptable range (46.0–56.6%), whereas the EE of CARκ-based microparticles was very low. The highest EE values were observed for the microparticles composed of the polysaccharide with the highest viscosity in the aqueous solution during the fabrication process, namely, SCMC and MALG.

**Figure 2:**
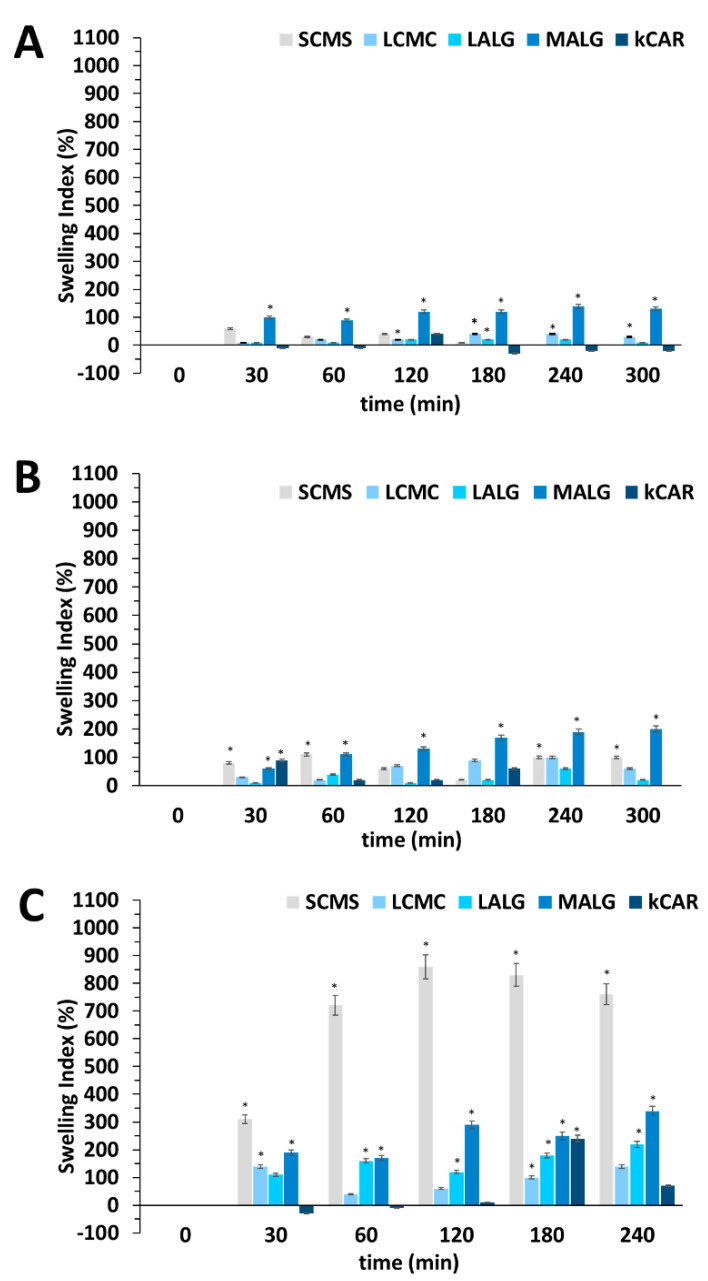
Comparison of the swelling indices of the CE-loaded polysaccharide microparticles in media at pH = 2 (**A**), pH = 7 (**B**), and pH = 10 (**C**). The results are presented as the means ± S.D. * The difference was statistically significant at *p* ≤ 0.05.

Physical stability in various media is an important feature to maintain during long-term storage; thus, the swelling behavior of the fabricated microparticles was examined. The swelling behavior could contribute to the pH-dependent release kinetics of the CE-loaded polysaccharide-based microparticles because the pore size in a polyelectrolyte matrix is altered as a result of its shrinkage or swelling induced by environmental conditions (pH, ionic strength, temperature, etc.), inhibiting uncontrolled payload release or facilitating controlled payload release. These phenomena have been observed for hydrogels; therefore, xerogels (dried microparticles) were first rehydrated within an aqueous environment. Each polymer (ALG_LV_, ALG_MV_, CMC_90_, CMC_250_, and CARκ) displayed very different abilities to absorb water due to the presence of different numbers of hydrophilic functional groups available for water molecules to form hydrogen bonds. Thus, during the initial rehydration step, CE-loaded microparticles absorbed water molecules in an amount typical for the particular core polysaccharide. The concentration of the core polysaccharide also reflects differences in the hydrodynamic free volume that contributes to the initial swelling of the microparticles. Thus, in order to reliably compare microparticle stability in different media, the calculated swelling indices (SIs) express the percentage of the medium retained in their structure after microparticle rehydration. Further changes in the mass of the CE-loaded polysaccharide microparticles that occurred after their rehydration, particularly in acidic and alkaline media, were evaluated, and the results of the swelling studies suggest a high physical stability of the microcarriers (Figure 2).

In the acidic environment, only MALG swelled extensively; the other microparticles swelled to a similar extent and remained stable during the experiment. An increase in the SI_pH2_ values of the microparticles was observed throughout the experiment because water diffuses more slowly in a dense polysaccharide matrix and the swelling of microparticles takes longer. The SI_pH2_ values of κCAR fluctuated, presumably due to the degradation of the core polysaccharide [6]. Under neutral conditions, LCMC, LALG, and κCAR remained in the similar same state as after rehydration. SCMC and MALG absorbed more medium than the other microparticles; however, the SI_pH7_ values remained rather low. In the alkaline environment, only SCMC and MALG showed significant swelling. Repulsive interactions between dense carboxyl groups in the ALG_MV_ and CMC_90_ chains resulted in matrix loosening and the absorption of a large amount of the medium. Moreover, the extensive degree of swelling observed for SCMC is probably due to the higher hydration capacity of trivalent ions used to crosslink the CMC-based microparticles than to divalent or monovalent ions used for stabilization of the other types of microparticles. A high concentration of CMC_90_ (7%) was used to form SCMC, and its stabilization required numerous crosslinks formed with aluminum ions. This phenomenon coupled with intensive matrix loosening due to internal repulsive interactions in alkaline medium led to very pronounced SCMC swelling. Additionally, in alkaline medium, unexpected and extensive swelling was observed for κCAR, but only at the 180 min time point of the experiment (SI_pH10_ = 240%), and the SI of κCAR subsequently returned to rather low value. This event was probably an artefact caused by non-sufficient centrifugation or filtration of the medium before the weighing procedure.

### 2.2. Effect of the Core Polysaccharide on the Stability of the Cranberry Extract over Time

**Figure 3:**
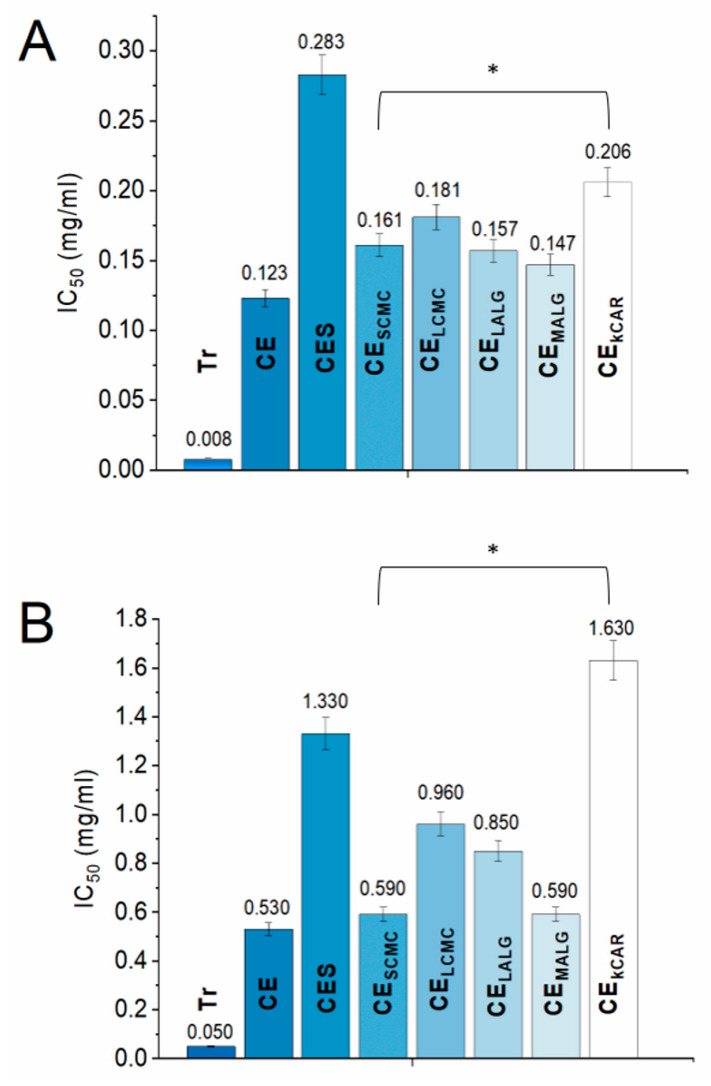
Comparison of the IC_50_ values of Trolox (Tr), cranberry extract inside the particles (CE), cranberry extract outside the particles (CES), and CE stored in the polysaccharide microparticles determined using the DPPH (**A**) and ABTS^+^ (**B**) assays. For both assays, the results are presented as the means ± S.D. * Statistically significant at *p* ≤ 0.05.

The chemical compositions of the selected polysaccharides significantly affected their ability to function as a separating barrier during storage. The M/G ratio of ALGs, the DS of CMCs, and the density of the sulfonate groups in CAR affect the formation of crosslinks in a hydrogel. If more crosslinks are formed, the hydrogel is less porous and is able to more efficiently isolate the payload from the external environment. Therefore, the effects of the selected polysaccharides on the biological stability of CE over time were investigated. Two assays were performed to evaluate the antioxidant activity of CE, CES, and CE stored in the polysaccharide microparticles to examine the different mechanisms of action of the CE components, and the results are depicted in Figure 3. The 2,2′-azino-*bis* (3-ethylbenzothiazoline-6-sulfonic acid) (ABTS) method monitors the activity resulting from the transfer of a hydrogen atom to an oxidant [24], and the 2,2-diphenyl-1-picrylhydrazyl (DPPH) method involves electron transfer from an antioxidant to DPPH [24]. In the present study, Trolox was used as a positive control for both antioxidant assays, as the substance is considered to possess a very high antioxidant capacity; Trolox tends to exhibit a free radical scavenging activity that is up to eight times more effective than vitamin E [25]. Several studies have already confirmed the high antioxidant potential of large cranberry fruit extracts, and the results presented in this paper are consistent with the results reported previously in the literature [17,18]. After 18 months of storage under ambient conditions (temp. 20–32 °C, atmospheric pressure), changes in the saccharide and phenolic compositions (expressed as gallic acid equivalents (GAEs)) of the plant extract (Table S1, Supplementary Materials) were observed. CE, CE stored in polysaccharide microparticles (CE_SCMC_, CE_LCMC_, CE_MALG_, CE_LALG_, and CE_kCAR_), and CES exhibited changes in the free radical scavenging activity in a dose-dependent manner, which was confirmed by the results obtained using both methods. The activity of the extracts recorded in the ABTS assay was slightly lower than the activity recorded in the DPPH assay, regardless of the type of extract (Figure 3). This slightly lower activity was probably caused by the unfavorable aqueous environment for CE created by the ABTS method. Comparing the antioxidant properties of CE and CES, a closer examination of CES revealed that the changes in the chemical composition negatively affected the biological activity of large cranberry fruit extract. The IC_50_ values of the radical scavenging activity (RSA) of CES measured using the DPPH and ABTS methods were significantly reduced compared to the IC_50_ values of CE, which suggests a destabilization of anthocyanin, probably due to the increased temperature, increased pH (Table S1), light, and presence of oxygen during storage. An environment with an elevated pH value increases the instability of anthocyanins and facilitates their conversion into equilibrium forms that are prone to oxidation and degradation [26]. Moreover, the antioxidant activity expressed as the IC_50_ of CE stored in the polysaccharide microparticles was 1.3–1.7 times lower in the DPPH assay and 1.1–1.8 times lower in the ABTS assay compared to CE. However, these values were still much higher than the value for the antioxidant activity of CES (Figure 3).

This reduced antioxidant potency of CE stored in microparticles was expected, as the stability of the CE compounds (in particular phenolics) is reduced when the extract is subjected to technological processes. Additionally, the rate of RSA differed for CE, CE stored in the microparticles, and CES, and the graphs are shown in Figure S3, Supplementary Materials. The trends of the antioxidant activities observed for CE stored in the microparticles were similar in both tests. The scavenging rate of CE stored in the microparticles followed the order of CE_SCMC_ > CE_MALG_ > CE_LALG_ > CE_LCMC_ > CE_kCAR_. In both antioxidant assays, the IC_50_ values of CE_SCMC_ and CE_MALG_ were similar to the IC_50_ of CE, indicating that the composition of CE was not significantly altered during storage. An exception was CE released from κCAR, where the activity of CE_kCAR_ measured in the ABTS test was 20.1% less than the activity of CES. Active compounds were generated in κCAR during the encapsulation process at high temperatures, most likely due to the degradation of CE. Overall, the IC_50_ values of CE_κCAR_ exhibited the greatest deviation from the IC_50_ of CE. Based on these results, the ALG- and CMC-based microparticles effectively protected the CE components, limiting their alteration and avoiding the deterioration of their biological activity during the 18-month evaluation. The microparticles protect the payload because they may reduce the risk of direct contact of the CE with air and humidity. Additionally, the chosen encapsulation strategy enabled the qualitative incorporation of CE into the polysaccharide particles, which confirmed the applicability of the used technique. The polysaccharides used as the core polymer for the encapsulation of CE helped preserve its stability, maintain its quality, and prolong its shelf life. Interestingly, 7.9 g/kg of MALG should be applied to achieve a similar antioxidant effect as Trolox administered orally for therapeutic purposes (50 mg/kg dose for rats) [25]. Nevertheless, Trolox is not currently used as a typical therapeutic agent.

### 2.3. Effect of the Core Polysaccharide on the In Vitro Release Behavior of Cranberry Extract

**Figure 4:**
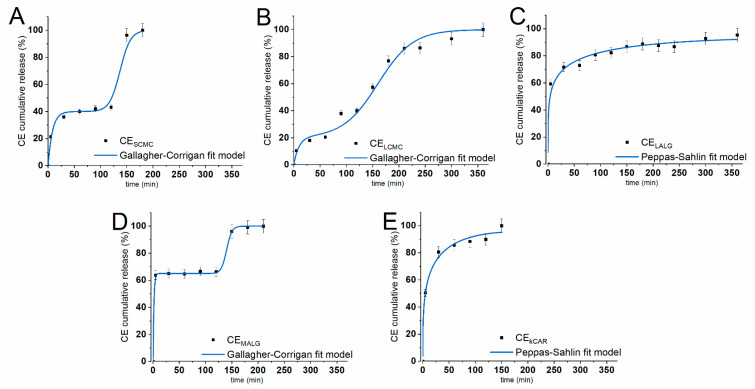
In vitro cumulative release profiles of CE from the polysaccharide microparticles (dots) (**A**) SCMC, (**B**) LCMC, (**C**) LALG, (**D**) MALG, and (**E**) κCAR) under conditions simulating the stomach (0–120 min) and intestines (121–360 min). Solid lines correspond to CE release profiles that best fit the selected kinetic models, namely, the Gallagher–Corrigan and Peppas-Sahlin models. The results are presented as the means ± S.D.

The procedure adopted for the CE in vitro release studies allowed us to accurately reproduce the conditions of the digestive system and study how the physiological conditions of the stomach and intestines modulate the release of the payload from the fabricated microparticles. This procedure [27] was specifically established to study the abilities of hydrogel polysaccharide-based microparticles to protect their payload under conditions simulating the gastrointestinal environment. The different behaviors of the polysaccharides in the changing conditions of the gastrointestinal tract are related to the consequences of the protonation and deprotonation of the functional groups in their structures—carboxylic acid groups in ALGs and CMCs, hydroxyl groups in ALGs and κCAR, and sulfonate groups in κCAR. Additionally, the presence of numerous ionizable functional groups in their chains leads to varying sensitivity to external pH. In vitro release studies were conducted under conditions simulating the upper (simulated gastric fluid (SGF); pH = 2.0) and lower (simulated intestinal fluid (SIF); pH = 7.5) parts of the gastrointestinal tract in terms of fluid composition, pH value, ionic strength, temperature, agitation, and time of residence. The release experiments were performed with dried, newly prepared CE-loaded microparticles that had not been subjected to prior storage. Each type of microparticle exhibited a different release behavior due to the response of the core polysaccharide to the changing environment (Figure 4).

Both ALG-based microparticles exhibited a burst release of the payload that lasted for 5 min for MALG and 30 min for LALG. Interestingly, the ALG-based microparticles exhibited a different release pattern in SGF, and the particles released different amounts of CE during this stage of the experiment, which exceeded 80% of the initial loading for LALG and was approximately 67% for MALG. The CMC-based microparticles also exhibited various release patterns. For LCMC, a burst release lasting approximately 30 min was observed, whereas a slow and steady CE release was observed for LCMC. Finally, both types of CMC-based microparticles released approximately 40% of the initial payload within 120 min in SGF. The κCAR released approximately 90% of CE under the gastric conditions, presenting a similar burst effect as LALG. All the microparticles, except for LCMC, exhibited a burst release during the initial stage of the SGF experiment (5–30 min), followed by a significantly slower release in SGF for the remaining time. Additionally, a plateau was noticed after 90 min and 5 min for the SCMC and MALG microparticles, respectively.

The release behavior of the studied hydrogels in SGF resulted from the properties of the polysaccharide matrix. Burst release effects are caused by the initial release of the payload molecules entrapped close to the particle surface. As the polymer structure changes in the acidic environment, the internal arrangement of the matrix also changes. In an acidic environment, carboxyl residues become protonated; therefore, the repulsion forces are reduced, leading to the shrinkage of the microparticle matrix [4]. The size of the microparticle pores decreases; thus, mass transfer is hindered and CE release is prevented. This phenomenon was best exemplified by MALG; the particles were the smallest and thus the medium quickly penetrated the microparticle matrix, causing matrix shrinkage and the inhibition of CE release within the first 5 min of the experiment. For SCMC, LALG, and κCAR, shrinkage of the matrix also occurred but proceeded more slowly due to the larger amount of matrix material (Table 1). Nevertheless, the burst release effect was enhanced by structural defects of the delivery devices, such as cracks or very large pores [28]. These defects probably caused the release of large amounts of CE from LALG and κCAR, and thus even the shrinkage of the matrix was not able to prevent the release of the payload.

When the particles were transferred into SIF, the release behavior changed. The polysaccharides used as the building material for the synthesized microparticles responded to moderately alkaline conditions (pH = 7.5). The loss of the hydrogen balance and the change in ionic strength weakened the structure of the microparticles. Most microparticles swelled slightly (Figure 2) due to internal electrostatic repulsive interactions between partially negatively charged carboxyl groups within the polysaccharide chains. Burst release was again observed for SCMC and MALG. Within the first 30 min of the SIF experiment, SCMC and MALG released approximately 96% of the payload, and the value reached 100% release after another 30 and 60 min, respectively. The remainder of the CE encapsulated in κCAR, which was probably located close to the center of the microparticles, was released in SIF at a slightly faster rate than in SGF due to the expansion of the matrix. CE release from LALG in SIF occurred at a much slower rate than in SGF, but it occurred in a steady manner. The most interesting release pattern in SIF was observed for LCMC, because the release was steady and lasted for almost 4 h—the amount of time the particles would spend in the intestines. During the experiment, the payload was completely released from the polysaccharide microparticles. The amount of CE detected in the release media was similar to the encapsulated amount, regardless of whether the release lasted for 150 min (for κCAR) or more than 6 h (for LALG).

The SCMC, LCMC, and MALG particles exhibited the desired release pattern that was tailored according to our expectations. These microparticles retained CE within their structure in the SGF experiment, indicating that they were able to transport the payload to the intestines and allow its absorption into the bloodstream. Although SCMC and MALG rapidly released their payload during the SIF experiment, LCMC showed a sustained, prolonged release behavior. LALG and κCAR most likely will be unable to transport a significant amount of CE to the lower parts of the digestive system; however, they successfully protect drugs and deliver them to the stomach, e.g., to help treat bacterial infections or ulcers.

The patterns of CE release from the polysaccharide microparticles were fitted to two kinetic models, namely, the Peppas-Sahlin [29] and Gallagher-Corrigan [30] models. These models were selected from multiple available models because they adequately describe the different release patterns by considering the behavior of the microparticles during the experiment conducted in two environments. The kinetic models were fitted to the experimental release data in SGF (0–120 min) and SIF (120–360 min), and the results of the best fits are shown in Figure 4. The model parameters, together with the correlation coefficient (R^2^), are presented in Table 1. The best fit model was selected based on its higher R^2^ value for the given release profile, and the values ranged from 0.969 to 0.999. The Peppas-Sahlin model is a power-law kinetic model that investigates whether the diffusion or erosion mechanism drives the release of the payload from the delivery system. The k_PS1_/k_PS2_ ratio should be calculated to determine which mechanism is dominant. The release patterns of LALG and kCAR were precisely described by the Peppas-Sahlin equation, and the mechanism of the CE release behavior was mainly characterized by diffusion, as the k_PS1_/k_PS2_ ratio was >1 for both types of particles [31]. This finding confirms the value of the m coefficient, which is <0.43 for both types of microparticles and corresponds to the n coefficient in the Korsmeyer-Peppas model, confirming the Fickian diffusion phenomena [32]. The Gallagher-Corrigan model is sufficient for describing diverse release behaviors that depend on the changing conditions, e.g., environmental conditions. SCMC, LCMC, and MALG clearly exhibited a two-stage CE release pattern. The release in SGF that occurred prior to matrix shrinkage and was dominated by the CE located close to the surface of the microparticles was considered the first stage. The second stage included the release in SIF when the matrix polysaccharides underwent relaxation. Therefore, the selected kinetic model is adequately suited to describe the release patterns of SCMC, LCMC, and MALG.

## 3. Materials and Methods

### 3.1. Materials

Organic solvents (ethanol, hexane) were purchased from Avantor Performance Materials (Gliwice, Poland). Low viscosity sodium alginate (ALG_LV_) (8 mPa·s, 1% in H_2_O at 25 °C; M/G ratio ~0.80; molecular weight (Mw) ~230 kDa) [33], medium viscosity sodium alginate (ALG_MV_) (11,130 mPa·s, 2% in H_2_O at 25 °C; M/G ratio ~1.56; Mw = 80–120 kDa) [34], sodium carboxymethyl cellulose Mw = 90 kDa (CMC_90_) (85 mPa·s, 4% in H_2_O at 25 °C; degree of substitution (DS) = 1.00) [35], carboxymethyl cellulose Mw = 250 kDa (CMC_250_) (640 mPa·s, 4% in H_2_O at 25 °C; DS = 0.79), κ-carrageenan (CAR_κ_) (16 mPa·s, 0.3% in H_2_O at 25 °C; 430 kDa) [36], Tween 80, Span 80, pepsin, bile salts, 2,2-diphenyl-1-picrylhydrazyl radical (DPPH·), 2,2′-azino-*bis* (3-ethylbenzothiazoline-6-sulfonic acid) (ABTS), and 6-hydroxy-2,5,7,8-tetramethylchroman-2-carboxylic acid (Trolox) were purchased from Sigma-Aldrich (Poznan, Poland). Liquid paraffin was obtained from Aflofarm (Pabianice, Poland).

### 3.2. Preparation of the Polysaccharide Microparticles

**Scheme 1:**
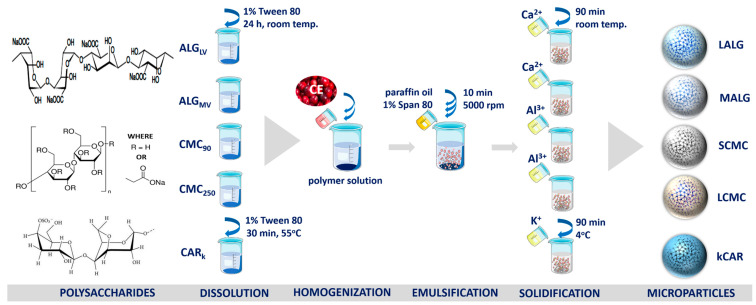
Schematic illustrating the procedure used to prepare polysaccharide microparticles loaded with CE. A detailed description is provided in Section 3.2.

ALG_MV_, ALG_LV_, CMC_250_, and CMC_90_ were dissolved in distilled water containing 1% Tween 80 to produce the chosen polymer concentration (Table 1). The CARκ solution was prepared analogously, except that the polymer was dissolved at 55 °C (Scheme 1). Then, large cranberry (*Vaccinium macrocarpon* Aiton) fruit extract (CE) (a detailed description of the CE preparation is included in Section S1.1 and Figure S1, Supplementary Materials) was added to the solution and carefully homogenized (3 min, 5000 rpm) (Ultra-Turrax T25, IKA, Wilmington, NC, USA) to obtain a finely dispersed suspension with a payload-to-polymer weight ratio of 2:3 (*w*/*w*). The resulting mixture was emulsified with liquid paraffin containing 1% Span 80 (10 min, 5000 rpm) (Ultra-Turrax T25, IKA) at a volume ratio of 1:4 (*v*/*v*). Next, the sodium alginate emulsions were transferred to 0.2 M CaCl_2_, the carboxymethyl cellulose emulsions were transferred to AlCl_3_ (1:4, *v*/*v*), and both were stirred for 90 min at room temperature. The κ-carrageenan emulsions were transferred to 2 M KCl and stirred for 90 min at 4 °C. Subsequently, the particles were collected, washed carefully with 1% Tween 80, and repeatedly washed with distilled water (including dialysis (MWCO 12–14 kDa)) to remove residues of unreacted substances, and finally dried at 4 °C for 48 h. The resulting particles, namely, SCMC, LCMC, LALG, MALG, and κCAR (refer to Table 1 and Scheme 1), were stored under ambient conditions for 18 months.

### 3.3. General Characterization of the Polysaccharide Microparticles

The encapsulation efficiency (EE) was determined by directly evaluating the amount of CE loaded into the microparticles. The CE-loaded microparticles were processed into a fine powder with a mortar and suspended in ethanol (1:25, *w*/*v*) for 24 h with intense agitation at room temperature. Then, the mixtures were filtered to remove any remaining solids and centrifuged (5000 rpm, 10 min) (Spectrafuge Mini Centrifuge, Labnet International Inc., Cary, NC, USA), and the absorbance was measured at λ = 287 nm (U-2900, Hitachi, Ibaraki, Japan). The *EE* was calculated using the following equation:

(1) $$EE\;=\frac{m_{s}}{m_{0}}\;\times 100\%$$

where *m*_0_ represents the initial mass of CE used for the encapsulation process and *m_S_* represents the amount of CE encapsulated in the microparticles. Notably, *m_S_* was estimated using an appropriate calibration curve with CE as the standard. The CE-loaded microparticles were first manually transferred onto double-sided adhesive tape to estimate the size, shape, and surface morphology. Subsequently, the microparticles were analyzed using a scanning electron microscope (SEM) (JSM-6601LV, JEOL, Tokyo, Japan) at 30–1440× magnifications and an accelerating voltage of 5–10 kV. The size of the microparticles was reported as the average particle diameter of at least 100 particles. The swelling studies were conducted in media with different pH values as follows: pH = 2.0 (10 mM HCl), pH = 7.0 (distilled H_2_O), pH = 10 (0.01 mM NaOH). The CE-loaded microparticles were suspended in the selected medium (1:25, *w*/*v*) and stirred for 15 min at room temperature to rehydrate the particles. Then, the mixture was centrifuged and filtered (5000 rpm, centrifuge tube filters, cellulose acetate membrane, 0.45 µm pore size) (Spectrafuge Mini Centrifuge, Labnet International Inc.) for 90 s, and the particles were weighed. Subsequently, the microparticles were suspended in the selected medium again, and the experiment was continued by stirring the suspension at room temperature. At selected time intervals, the mass of the microparticles was measured. The swelling index (*SI*) was calculated using the following equation:

(2) $$SI\;=\frac{m_{T}-m_{RH}}{m_{RH}}\;\times 100\%$$

where *m_T_* represents the measured mass of the microparticles at the selected time point, while *m_RH_* represents the mass of the rehydrated microparticles [21].

### 3.4. Antioxidant Activity Studies

CE and CES (CE stored at room temperature for 18 months) were studied as received. The CE-loaded microparticles were first minced into a fine powder and suspended in ethanol (1:100, *w*/*v*) for 24 h with intense agitation at room temperature. Then, the obtained mixtures were centrifuged (10,000 rpm, 5 min), filtered (syringe filter, nylon membrane, 0.45 μm pore size), and finally the CEs removed from polysaccharide microparticles were lyophilized to obtain CE_SCMC_, CE_LCMC_, CE_MALG_, CE_LALG_, and CE_κCAR_. For the analysis of the antioxidant activity of CE, CE encapsulated in polysaccharide microparticles (CE_SCMC_, CE_LCMC_, CE_MALG_, CE_LALG_, and CE_κCAR_) and CES were dissolved in ethanol to obtain the stock solutions with the concentrations of 5 mg/mL, 2 mg/mL, and 10 mg/mL. Their scavenging effect was evaluated with the DPPH [37] and ABTS [38] methods. Trolox served as the positive control, while ethanol served as the negative control. The absorbance of the samples was measured at λ = 517 nm for the DPPH method and λ = 734 nm for the ABTS method using a spectrophotometer (U-2900, Hitachi). The radical scavenging activity (*RSA*) was calculated using the following equation:

(3) $$RSA\;=\;\frac{A_{0}\;-\;A_{S}}{A_{0}}\;\times 100\%$$

where *A*_0_ represents the absorbance of the sample without the radical scavenger and *A_S_* represents the absorbance of the sample containing the radical scavenger at a particular concentration. The IC_50_ value was calculated using the Origin Pro 9.0 software interpolation function based on the calibration curve of RSA as a function of the concentration of the studied extract.

### 3.5. In Vitro Release Studies

The microparticles were immersed in simulated gastric fluid (SGF) (0.9% NaCl and 0.3% pepsin; pH = 2.0) (1:100, *w*/*v*) for 120 min with gentle agitation at 37 °C. Subsequently, the microparticles were filtered, thoroughly washed with distilled water, transferred to simulated intestinal fluid (SIF) (0.65% NaCl, 0.08% KCl, 0.02% CaCl_2_, 0.14% NaHCO_3_, and 0.3% bile salts; pH = 7.5) (1:100, *w*/*v*), and gently agitated for an additional 4 h at 37 °C [27]. At predetermined time points, samples were withdrawn from the release medium (1:30, *v*/*v*), and the release medium was refilled with fresh SGF or SIF. Subsequently, the withdrawn samples were thoroughly mixed with ethanol (1:1, *v*/*v*). Then, the samples were centrifuged (10,000 rpm, 5 min) and filtered (syringe filter, nylon membrane, 0.45 μm pore size), and the absorbance of the samples was measured (U-2900, Hitachi). The curves of release kinetics were constructed from the wavelength (λ = 287 nm) with the maximum absorbance observed in UV-Vis spectra for CE and CES dissolved in ethanol (C = 2 mg/mL) (Figures S4 and S5, Supplementary Materials [39,40,41,42]). The cumulative release of *CE* was calculated using the following equation:

(4) $$CR_{CE}\;=\frac{m_{RE}}{m_{t}}\;\times 100\%$$

where *m_RE_* is the amount of *CE* released to each sample at various time points and *m_t_* is the total encapsulated mass of *CE*. The value of *m_RE_* was estimated using an appropriate calibration curve with *CE* as the standard. The release profiles are reported as the cumulative release of *CE* as a function of time. Additionally, the data from the in vitro release experiment were fitted to the Peppas-Sahlin [29] and Gallagher-Corrigan [30] models using Origin Pro 9.0 software to estimate the kinetic parameters of the *CE* release behavior. The Peppas-Sahlin model is described using the following equation:

(5) $$\frac{M_{t}}{M_{\infty }}\;=\;k_{PS1}\times t^{m}+\;k_{PS2}\times t^{2m}$$

where *k_PS_*_1_ represents the diffusion rate constant, *k_PS_*_2_ represents the erosion rate constant, and *m* represents the diffusion exponent. The Gallagher-Corrigan model is described using the following equation:

(6) $$\frac{M_{t}}{M_{\infty }}\;=M\left[1-e^{-k_{GC1}t}\right]+\left(M_{\infty }+M+\;M_{SGF}\right)\frac{e^{k_{GC2\;\left(t-tmax\right)}}}{1+\;e^{k_{GC2}\left(t-tmax\right)}}$$

where *M* represents the amount of *CE* released in *SGF*, *k_GC_*_1_ and *k_GC_*_2_ represent the stage kinetic constants, and *t_max_* represents the time point of the fastest *CE* release into SIF.

### 3.6. Statistical Analysis

Statistical analyses of the collected data were performed using Microsoft Office Excel 2019 and Origin Pro 2019. Each experiment was performed at least three times. The results are reported as mean values with standard deviations (±S.D.). The statistical significance of the differences between mean values and the control (untreated) group was evaluated by Student’s *t*-test. A *p*-value ≤ 0.05 was considered statistically significant.

## 4. Conclusions

Five types of polysaccharides with various structures (alginates (ALG_LV_, ALG_MV_), carboxymethyl celluloses (CMC_90_, CMC_250_), and κ-carrageenan (CAR_κ_)) were successfully used to encapsulate large cranberry fruit extract (CE). Thorough characterization of the fabricated microparticles confirmed their spherical shape with a diameter of approximately 50 µm. Studies of the microparticle performance revealed that SCMC, LCMC, and MALG released their payload with the desired release pattern under gastrointestinal conditions in vitro, suggesting that the polysaccharides securely deliver their payload to the intestine. Additionally, LALG, MALG, SCMC, and LCMC are promising candidates for the efficient long-term protection of the payload to prevent a loss of their biological properties, as CE retained its antioxidant activity during encapsulation and over the next 18 months.

Overall, the advantage of using polysaccharides to form microparticles for application as storage vehicles for unpreserved phytochemicals is that polysaccharides protect their payload from deterioration, which significantly improves its stability, even under ambient conditions. In addition, the results of this study will allow researchers to select the polysaccharides that are most compatible with CE and, as such, are most useful for the fabrication of a promising particle release platform that is responsive to gastrointestinal conditions and delivers and releases the payload to a selected part of the gastrointestinal tract. Therefore, the studied polysaccharides are considered efficient materials for the encapsulation, controlled delivery, and protection of phytochemicals applicable in several food or pharmaceutical products.

## Figures and Tables

**Table 1:** Composition, characteristics, and kinetic parameters generated from mathematical models of the experimental data obtained from the in vitro release of the CE-loaded polysaccharide microparticles. Bold values indicate the best fit to the kinetic models. * The results are presented as the means ± S.D.

Particle	Composition		EE * (%)	Size * (µm)	Peppas-Sahlin				Gallagher-Corrigan				
	Core Polysaccharide	C_core polysach_ (%)			*k*_PS1_	*k*_PS2_	*m*	*R* ^2^	*M*	*k*_GC1_	*k*_GC2_	*t*_max_	*R* ^2^
**SCMC**	CMC_90_	7.0	57.3 ± 1.3	48.4 ± 18	2.32	0.11	0.58	0.773	**40**	**0.12**	**0.10**	**137**	**0.982**
**LCMC**	CMC_250_	2.0	43.8 ± 0.9	54.4 ± 28	−2.39	2.74	0.32	0.949	**20**	**0.10**	**0.03**	**160**	**0.984**
**LALG**	ALG_LV_	3.0	56.6 ± 1.2	56.6 ± 26	**43.25**	**−5.00**	**0.23**	**0.969**	75	0.25	0.02	160	0.967
**MALG**	ALG_MV_	2.0	58.1 ± 0.9	46.0 ± 15	33.70	0.38	0.18	0.794	**65**	**0.60**	**0.20**	**140**	**0.999**
**κCAR**	CAR_κ_	2.5	28.0 ± 2.4	50.6 ± 21	**35.84**	**−3.36**	**0.32**	**0.983**	80	0.30	0.02	130	0.965

## Supplementary Materials

The following materials are available online, Figure S1. Schematic illustrating the procedure used to isolate the CE. Figure S2. FTIR spectra of the CE, CES, and CE-loaded microparticles (SCMC, LCMC, LALG, MALG, κCAR). Figure S3. Antioxidant activity of the cranberry extracts (CE, CES, and CE stored in the polysaccharide-based microparticles) towards DPPH^·^ and ABTS^+^, as reported as dependence of the RSA on the logarithmic function of the concentration. Figure S4. UV-Vis spectra recorded for CE and CES (C = 2 mg/mL) dissolved in ethanol. Figure S5. Calibration curve presenting the relationship between absorbance and the concentration of CE (in ethanol) released from the CE-loaded polysaccharide-based microparticles. The results are presented as the means ± S.D. Table S1: Characterization of the large cranberry fruit extracts CE and CES.
