# Supplementary material for: Alginate-, Carboxymethyl Cellulose-, and κ-Carrageenan-Based Microparticles as Storage Vehicles for Cranberry Extract
Source: Molecules. 2020 Sep 2;25(17):3998. doi: 10.3390/molecules25173998 (PMC7504800; doi:10.3390/molecules25173998)
Supplement: Supplementary file 1 [file molecules-25-03998-s001.pdf]

SUPPLEMENTARY MATERIALS

S.1. Methods

S.1.1. Preparation and characterization of the cranberry extract

Fruits of large cranberry (*Vaccinium macrocarpon* Aiton) were purchased from a local market (Poland), and the species was registered in the Wrocław University Botanical Garden (Poland) with voucher specimen no. 003011. Finely chopped large cranberry fruits were extracted twice with ethanol (1:10, w/v) for 6 h at 78°C. The extracts were combined and filtered, and the filtrate was further centrifuged (5000 RPM, 10 min) to remove solids. Subsequently, the volume of the fruit extract was reduced ca. ten-fold (ethanol was evaporated under reduced pressure) and extracted with hexane (1:1, v/v) for 6 h at 68°C. Afterwards, the fruit extract was separated from the hexane fraction, the remaining solvent was evaporated under reduced pressure, and the fruit extract was ultimately freeze-dried, yielding CE in the form of a thick dark-pink paste (Figure S1). The extraction yield (EY), which was calculated as the weight ratio of the obtained extract to used fruit, is presented in Table S1. Then, some of the CE was encapsulated in the polysaccharide microparticles, and the remaining CE was stored at room temperature for 18 months as CES. The saccharide contents of CE and CES were estimated with the phenolic-sulfuric acid method [39], while the phenol contents were estimated with the Folin-Ciocalteu method [40], and the results are presented in Table S1.

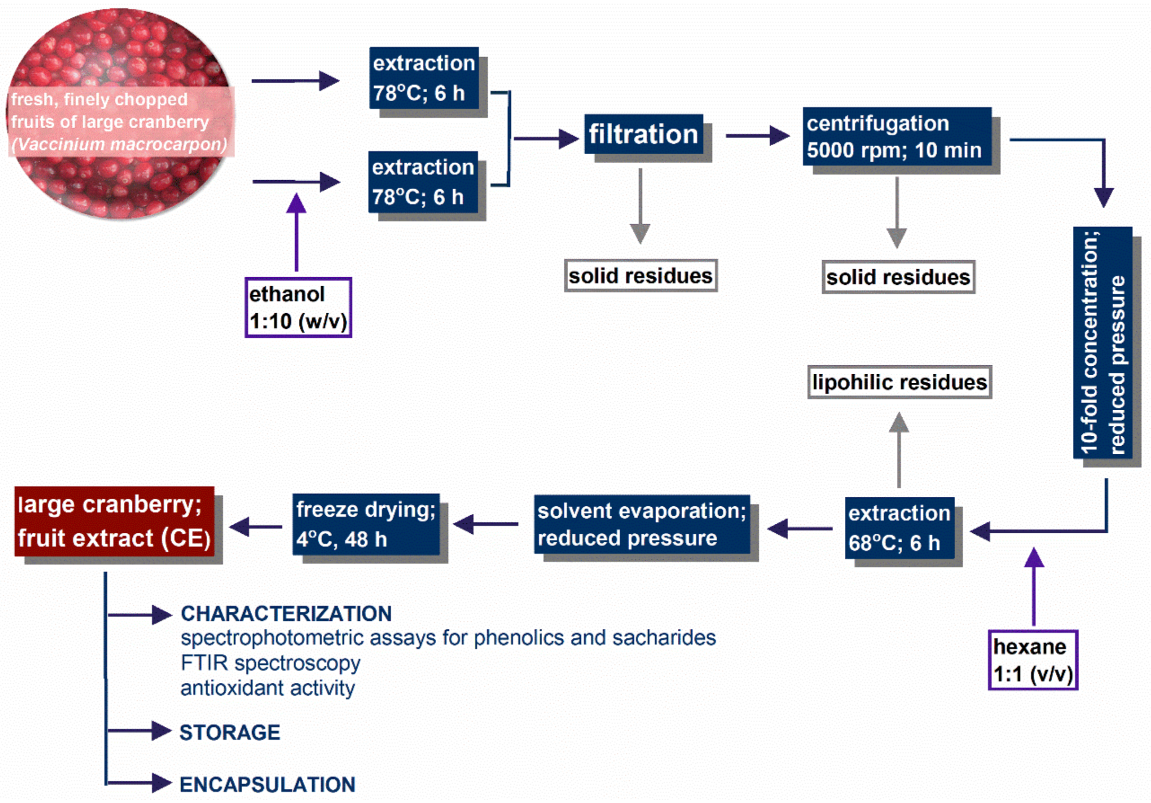

Figure S1. Schematic illustrating the procedure used to isolate the CE.

S.1.2. FTIR studies

CE and CES were analysed in the form in which they were isolated, while the microparticles were processed into a fine powder with a mortar prior to analysis. Fourier transform infrared (FTIR) spectra were recorded using an attenuated total reflectance (ATR) sampling accessory equipped with a diamond crystal on a Bruker VERTEX 70 V vacuum spectrometer (Bruker Optik GmbH, Germany). The

spectra were acquired in the range of 4000–400 cm^{-1} with 64 scans at a resolution of 2 cm^{-1} at room temperature.

S.2. Results and discussion

The results of the FTIR spectroscopy analyses of CE and CES are consistent with the literature [19, 41]. Based on the absorption peaks (Figure S2), the main components of CE and CES are confirmed to be organic acids, carbohydrates, phenolics and their glycosides: 3400–3200 cm^{-1} (-OH stretching vibration resulting from either saccharide or polyphenolic compounds), 2950–2900 cm^{-1} (asymmetric -C-H stretching vibration), ~1715 cm^{-1} (-C=O stretching vibration in the carbonyl groups of esters and carboxylic acids), an overlapped band at ~1400 cm^{-1} (-C=C- stretching vibration in the aromatic ring and symmetric COO- stretching vibration), ~1200 cm^{-1} (-C-O-C- stretching vibration in phenolic structures) and the wide, overlapped band at 1150–970 cm^{-1} (-C-O, -C-C- and C-OH in saccharide structures) [23]. In the CES spectrum, an additional signal at 2984 cm^{-1} attributed to the C-H stretching vibration in aldehyde groups appeared. Based on this finding, the carboxylic acid groups were deprotonated because the pH value of the extract increased slightly (from 2.20 to 2.77) over time (Table S1), indicating that these groups may have been converted to aldehydes. Saccharides can also undergo oxidation [42].

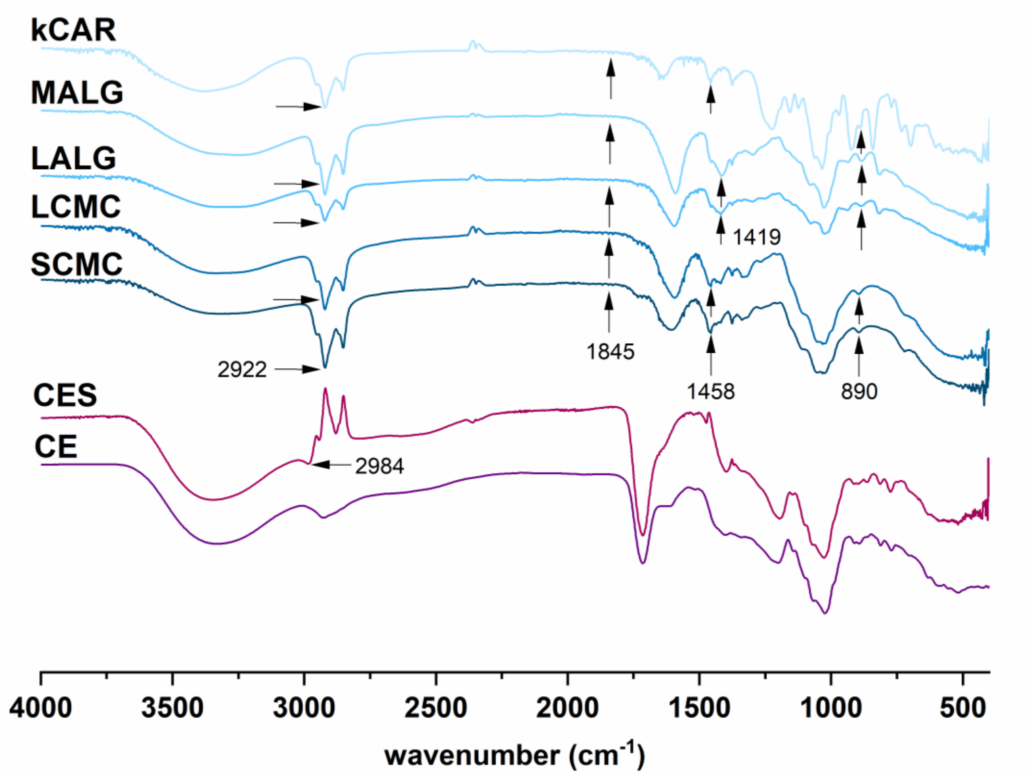

Figure S2. FTIR spectra of the CE, CES, and CE-loaded microparticles (SCMC, LCMC, LALG, MALG, κCAR).

Table 1S. Characterization of the large cranberry fruit extracts: CE and CES.

	EY (%)	GAE[1] (μM)	Saccharides (%)	pH
CE	39.2±1.4	566±11	39.5±3.5	2.20
CES	n.a.[2]	302±7	29.8±3.8	2.77

[1] GAE: gallic acid equivalents.

[2] n.a.: not applicable

Colorimetric analyses of the saccharide and phenolic contents in CES revealed 10% and 47% decreases, respectively, relative to CE, although the FTIR spectra of both extracts were very similar with only slight differences (Figure S2).

The spectra of the CE-loaded polysaccharide-based microparticles (Figure S2) were generally similar and exhibited signals that are typical for the core polysaccharides, as they have similar structures and constitute ≥ 85% of the loaded microparticles. Therefore, the following signals were resolved to confirm the successful encapsulation of the CE: the distinct characteristic peak at 2922 cm^{-1} associated with asymmetric stretching vibrations of C-H groups in aliphatic compounds present in the spectra of microparticles and CE; the very weak bands visible in the range of 1900 - 1750 cm^{-1} that are present in all microparticles spectra are due to overtones of aromatic compounds in the encapsulated CE; the signals in the range of 1458–1419 cm^{-1} are attributed to the C=C stretching vibrations of aromatic structures in phenolic compounds and shifted from lower values 1400 cm^{-1} for CE; and the signals at 890 cm^{-1} are attributed to β-glycosidic bonds [42].

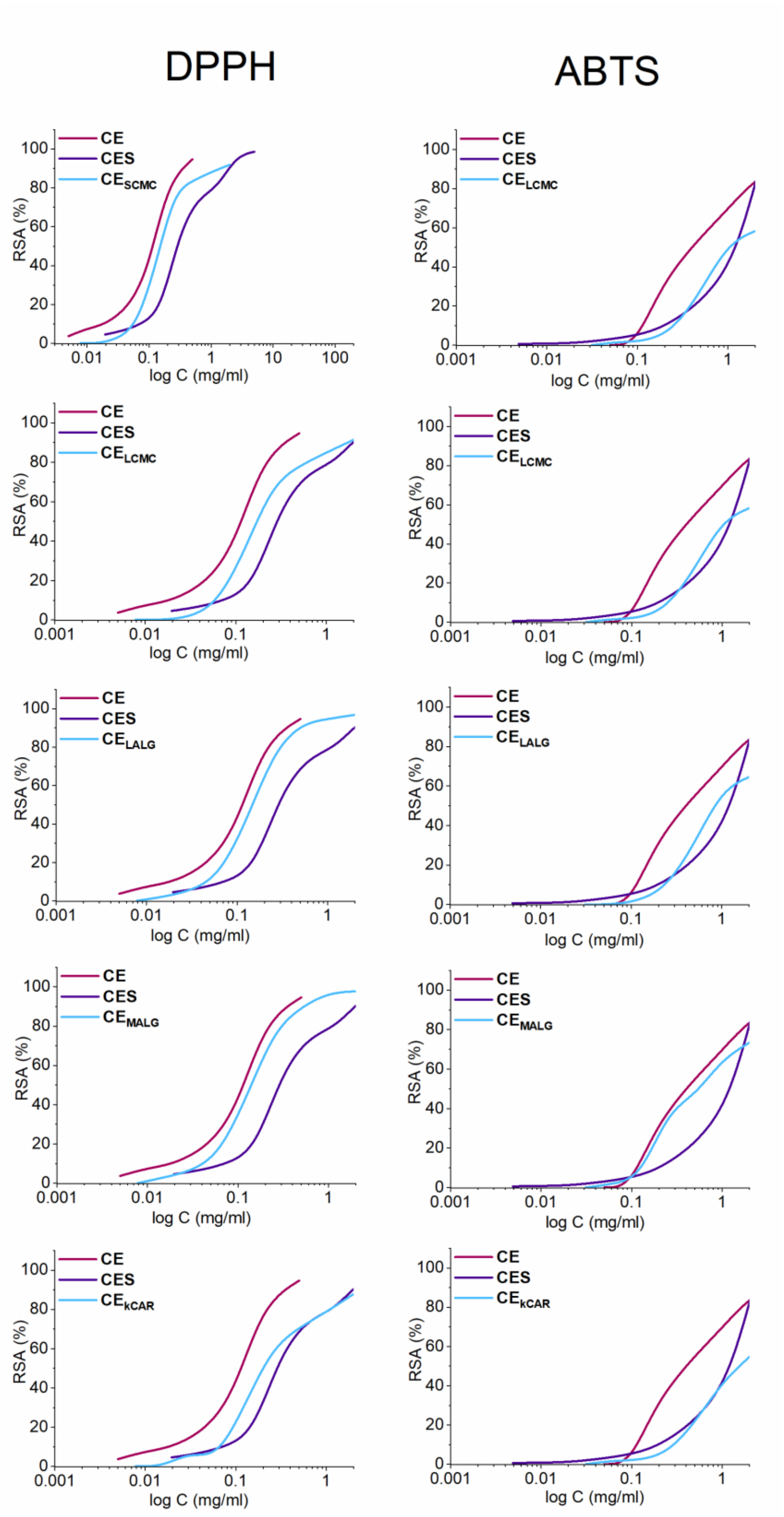

Figure S3. Antioxidant activity of the cranberry extracts (CE, CES, and CE stored in the polysaccharide-based microparticles) towards $DPPH^{\cdot}$ and $ABTS^{+}$, as reported as dependence of the RSA on the logarithmic function of the concentration.

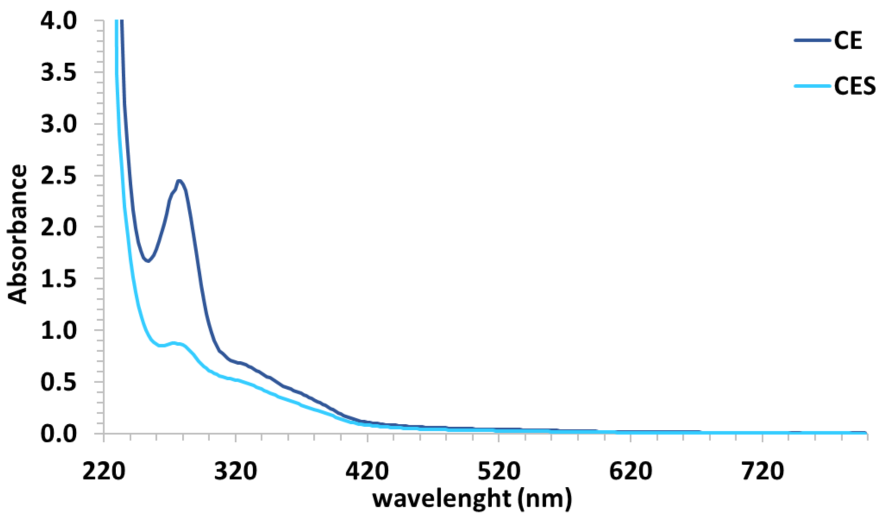

Figure S4. UV-Vis spectra recorded for CE and CES (C = 2 mg/ml) dissolved in ethanol.

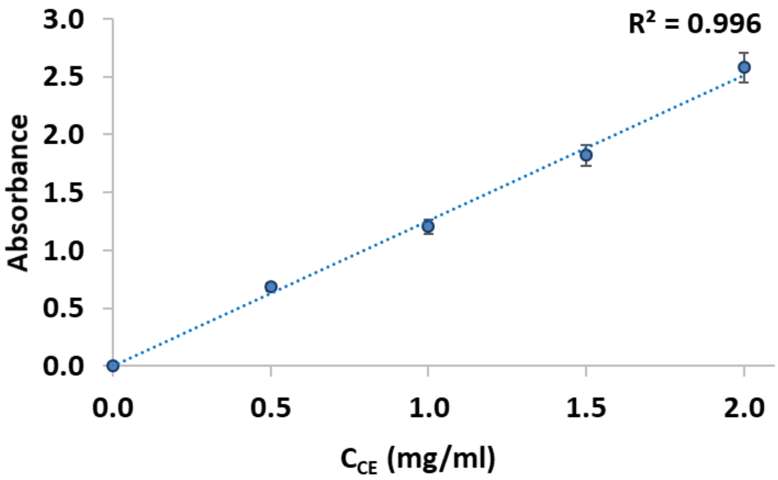

Figure S5. Calibration curve presenting the relationship between absorbance and the concentration of CE (in ethanol) released from the CE-loaded polysaccharide-based microparticles. The results are presented as the means ± S.D.
